# Considerations for state-imposed conditions on healthcare provider transactions

**DOI:** 10.3389/fpubh.2023.1220624

**Published:** 2023-08-16

**Authors:** Alexandra D. Montague, Robin L. Davison, Katherine L. Gudiksen, Jaime S. King

**Affiliations:** ^1^The Source on Healthcare Price and Competition, University of California College of the Law, San Francisco, San Francisco, CA, United States; ^2^Faculty of Law, University of Auckland, Auckland, New Zealand

**Keywords:** consolidation, conditions, merger, acquisition, consent decree, conditional approval, market failure, healthcare

## Abstract

The widespread consolidation of health systems, hospitals, and physicians has contributed to the high price of healthcare across the United States. While federal antitrust enforcers continue to play an important role in overseeing large mergers, acquisitions, and other consolidating transactions of major healthcare providers, state oversight over healthcare markets is essential to slow consolidation more broadly and address market failures across the country. State laws govern the scope of authority held by state attorneys general and other state agencies to receive notice of, review, and approve, conditionally approve, or block healthcare provider transactions, which can significantly impact the breadth and content of oversight. While blocking potentially anticompetitive transactions can help states maintain any competitive forces that remain in the market, in some situations, approving a transaction with conditions may be the best path forward. Applying conditions to transactions may allow state officials to oversee and govern the behavior of providers post-transaction while states pursue other legislative fixes. Although the use of conditions is a relatively common practice at the state level, little research has been done to explore their use among states. Following a search in all 50 states, this paper examines decisions from state officials imposing conditions intended to address the impacts of transactions on healthcare price, access, and quality and provides recommendations for the effective use of conditions moving forward.

## 1. Introduction

Unfettered consolidation among healthcare providers, including health systems, hospitals, and physicians, has deeply impacted how Americans receive health care and how much they pay for it. Most healthcare provider markets across the United States are now considered highly concentrated (1), and a majority of hospitals are associated with increasingly powerful health systems (2). Healthcare experts consistently find that highly concentrated healthcare provider markets are associated with higher prices, mixed quality outcomes, and reduced access to healthcare services (3), while other studies have shown that health systems with substantial market power can wield it across markets to engage in anticompetitive practices (4).

Addressing the existing market failures that plague most hospital markets in the U.S. will necessarily involve broad policy interventions to restrain high and rising healthcare prices and to protect access to affordable healthcare services (5). At the state level, these types of policies include initiatives that aim to more directly control healthcare costs such as creating cost-growth benchmarks, public options, affordability standards authorizing state insurance commissioners to reject contracts with excessive rate increases, direct price caps on out-of-network, in-network prices, or both, among others (6). Although a few states have attempted some of these policies and more are showing interest, mustering the political willpower to create and implement them takes time (6).

In the meantime, consolidation continues apace, and state regulators and enforcers tasked with overseeing mergers, acquisitions, and other consolidating healthcare provider transactions must decide whether and how to challenge these transactions. Generally, their options include letting the transaction proceed unimpeded, imposing conditions to dictate behavior post-transaction, or attempting to prevent the transaction from going through. Due to the highly concentrated nature of healthcare markets throughout the U.S., we argue that states should utilize the legal authority they have to block potentially anticompetitive healthcare transactions when possible. However, when blocking a potentially anticompetitive transaction is not an option because of resource constraints, legal limitations, political pressures, or because the transaction is truly in the public interest, imposing conditions on the transaction that dictate the healthcare providers’ behavior post-transaction, can serve as an important tool, allowing states time to pursue legislative fixes for any existing market failures.

Healthcare markets have consolidated substantially throughout the last 30 years due to permissive enforcement strategies at both the federal and state levels and a lack of alignment between antitrust guidelines and healthcare market practices (7). The federal antitrust agencies—the Federal Trade Commission (FTC) and the Department of Justice (DOJ)—are restricted by only receiving notice of large transactions due to the high reporting threshold of the Hart-Scott-Rodino Antitrust Improvements Act (8), limited resources to analyze and challenge a significant number of cases (9), and a significant burden to show that a transaction will substantially lessen competition under federal antitrust law (10). Furthermore, federal antitrust enforcement tends to focus on horizontal merger challenges rather than vertical, cross-market, or other types of consolidation, which commonly occur in healthcare (4). State entities tasked with overseeing healthcare transactions, including state attorneys general, state health agencies, and certificate of need (CON) programs, face similar resource limitations and are also constrained by the boundaries of their varying legal authorities to receive notice of, thoroughly review, and potentially block transactions (11).

When it comes to challenging problematic transactions, federal antitrust enforcers have expressed a preference for blocking anticompetitive transactions or imposing structural remedies, such as divestiture of any entities that create competition concerns, instead of relying on what are called conduct remedies, which permit the transaction to go through but include conditions that seek to prevent anticompetitive behavior (12). While various state entities have also successfully blocked healthcare provider transactions, we have found that state enforcers and regulators more often utilize conditions to manage, rather than trying to block, transactions that raise concerns (11). While the use of conditions can provide guardrails to ensure that healthcare providers behave in the public interest, the fact that these conditions are most often time-limited and only apply to the providers involved in the transaction suggests that their use cannot be relied upon as a long-term regulatory solution in most situations.

State officials may rely on conditions instead of blocking potentially anticompetitive transactions for a few reasons. First, states review and make decisions regarding a broader range of transactions than federal antitrust enforcers, including transactions that are either too small for federal antitrust scrutiny or are unlikely to harm competition in ways that form the basis of a convincing antitrust case (13). Furthermore, when state attorneys general consider proposed transactions under state or federal antitrust law, they may not have or wish to expend limited resources to litigate a case that does not fit the mold for a strong, traditional antitrust suit (14). Second, unlike federal antitrust enforcers, state attorneys general and various state agencies must often balance various, sometimes competing, priorities that extend beyond preserving competition when reviewing transactions, such as maintaining access to healthcare services (e.g., saving a financially failing hospital that provides essential services to a community), improving equitable access to care, protecting jobs, overseeing a hospital’s nonprofit obligations, and then weigh any claimed benefits and efficiencies from the transaction against the risks of adverse outcomes. Third, state officials can be statutorily limited by the factors they can consider in making their decision or by constraints on the type or size of the transactions they review. This means they may not have the power to block a transaction on competition grounds or do not have authority over certain transactions. Despite these dynamics, the widely held goals of protecting any remaining competition, controlling costs, preserving access, and improving equity cannot be achieved in the long run if consolidation continues unencumbered and without farther-reaching intervention to address pervasive market failures.

Whatever the reasons for placing conditions on a transaction, state enforcers and regulators must acknowledge and consider the serious potential drawbacks of allowing transactions to proceed in this way. Short-term conditions only dictate conduct momentarily, enabling healthcare providers to reap significant benefits from the accumulated market power after the conditions expire. As a result, harms from transactions are merely delayed, left to be dealt with by another administration, another agency, or often not at all. As one judge noted in rejecting a settlement proposed by the Massachusetts attorney general, imposing conditions on a problematic transaction is “like putting a band-aid on a gaping wound that will only continue to bleed (perhaps even more profusely) once the band-aid is taken off (15).” Further, even during the period in which conditions are imposed, the necessary regulatory oversight and monitoring of conditions to ensure compliance requires significant resources and expertise, which many states do not have the resources to consistently provide.

Although the use of conditions has been and continues to be a relatively common practice among states, little research has been done to explore their use at the state level. To examine the scope and usefulness of conditions, we searched for all available decisions by states that apply conditions on healthcare provider transactions. We reviewed over 80 decisions placing conditions on healthcare provider transactions from 12 states under varying legal authorities. This paper examines the array of conditions that state attorneys general and various state agencies have imposed on healthcare provider transactions over the past decade and offers considerations and recommendations for deciding whether—and how—to impose conditions.

Part I of this paper provides a brief overview of the various sources of legal authority available to state officials to review and condition approval of transactions. Part II provides a taxonomy of the types of conditions imposed through these different processes and their enforcement mechanisms. Lastly, Part III provides important considerations for state officials looking to impose conditions as a means to allow transactions to proceed. Often, there is no perfect solution when attempting to balance multiple facets of a healthcare market. While imposing conditions on transactions in some cases may be the best option, it should be done with specific goals in mind and an understanding of the consequences of permitting transactions to proceed with time-limited requirements.

## 2. Overview of state healthcare transaction conditional approval authority

The statutory authority to review and subsequently approve, conditionally approve, or deny healthcare transactions varies notably among the states (11). The actions state officials take and the types of conditions they impose will often depend largely on the kind of legal authority they have to review various types of transactions. This authority also dictates whether state officials must go to court or can approve transactions outside of court through an administrative process (16). For instance, a state attorney general that has challenged a transaction under antitrust law may enter into a court-approved negotiated settlement, known as a consent decree, with the transacting entities to impose conditions on the transaction. Whereas, when a state agency (or the attorney general in some states) has a form of administrative review authority, they can impose conditions on a proposed merger, which we refer to as a conditional approval. By analyzing the laws undergirding these different processes, we found that the legal boundaries of the approval authority often dictate whether state officials can impose conditions and the types of conditions imposed. For example, if an attorney general’s authority to review nonprofit hospital transactions is limited to statutorily delineated factors that focus on the protection of charitable assets, it is unlikely that they can impose conditions aimed at protecting broader competition concerns, like imposing price restraints. As agencies are bound by their legal authority, not all conditions are available for every type of approval process.

In every state, attorneys general have the authority to bring suits as *parens patriae* on behalf of the citizens of their state under state antitrust laws, federal antitrust laws, or both, as well as under consumer protection laws (16). Different states have invoked this authority to varying degrees in the healthcare context. For example, the Pennsylvania attorney general has challenged hospital transactions in court under antitrust laws that ended with consent decrees between the attorney general and the transacting healthcare providers that impose conditions mainly addressing competitive harms arising from the deal (17, 18).

In addition to enforcing antitrust laws, state attorneys general are also charged through statute and common law with the duty to oversee nonprofit and charitable organizations, to ensure the organizations fulfill their fiduciary duties, that charitable assets are appropriately managed and spent, and that the organizations are fulfilling their charitable mission (19). When it comes to transactions involving nonprofit hospitals specifically, several states have statutorily outlined administrative review and approval processes that permit the attorney general to oversee and place conditions on these transactions when necessary. For example, Connecticut’s attorney general reviews and has conditional approval authority over nonprofit hospital conversions, meaning instances where a nonprofit hospital enters into an agreement to transfer a material amount of its assets or operations to a for-profit entity (20). The New Hampshire Director of Charitable Trusts, housed within the attorney general’s office, reviews and has approval authority over transactions involving healthcare charitable trusts, including nonprofit hospitals (21). In the healthcare context, charitable trusts are often established by donors to fund healthcare services, charity care, or similar purposes (22). Attorneys general are responsible for ensuring these trusts are used in accordance with the donors’ original intent, even after changes in the organization or structure of the hospital to which the charitable trust was given (22). California also has a process in place for reviewing and approving transactions involving a nonprofit hospital (23). The statutes governing this process provide the attorney general with a notable amount of discretion to review these transactions under a wide range of factors (23). Considering that over half of the hospitals in the U.S. are nonprofit and nonprofit hospitals owe special obligations to the communities they serve, this type of review provides crucial oversight over a significant number of transactions (24). Further, in some instances, nonprofit oversight authority will allow state attorneys general to impose conditions related to competition and market function (25).

A few states grant authority to their department of health or similar state health agency to conduct reviews and approve, conditionally approve, or deny transactions, with some states outlining processes where the department of health conducts concurrent reviews of transactions with the state attorney general (13). For example, under the Rhode Island Hospital Conversions Act, the Rhode Island Department of Health and the Rhode Island attorney general conduct concurrent reviews of healthcare provider transactions (26). The Act assigns differing review criteria to the attorney general and department of health, as well as different sets of criteria depending on whether the transaction is solely among nonprofit hospitals or whether for-profit entities are involved. While Rhode Island attorney general’s review and approval focuses on whether the transaction is fair, whether the hospital board acted appropriately, and whether the transaction is proper under Rhode Island’s Nonprofit Corporation Act, Charitable Trust Act, and Antitrust Act, the Department of Health’s review and decision focuses on how the transaction will impact the community and access to affordable care (27).

Certificate of Need (CON) programs can also provide the statutory authority to review and approve healthcare provider transactions. CON programs are regulatory mechanisms that require hospitals and health systems to apply for permission from the state agency in charge of the CON program before making significant capital expenditures or changes to their facilities, services, or equipment (28). Although 35 states currently have CON programs, these programs vary from state to state, both in the activities that trigger review and the types of entities they regulate (29). Notably, not all CON programs grant the oversight agency authority over provider transactions, such as mergers and acquisitions, or permit the agency to impose conditions (29). Some states though, such as Connecticut and Massachusetts, do have broader CON programs that can serve as a means to review transactions and examine the impact of the transaction on the healthcare landscape and approve, condition, or deny the transaction (30, 31). Although there is a widely held belief that CON programs should be repealed because they create anticompetitive barriers to entry into hospital markets, they can potentially be useful as a means to receive notice of, review, and make decisions about potential transactions or expansions by a dominant health system that may have a broad impact on health care delivery throughout the state (32).

Beyond CON programs, three states have created independent state agencies tasked with overseeing healthcare markets, including providing in-depth reviews of a wide range of proposed healthcare transactions as well as monitoring other state price and transparency policies (33). In 2012, Massachusetts’ created the Health Policy Commission (HPC), which reviews a wide range of transactions and submits reports advising the Massachusetts’ attorney general and the Massachusetts Department of Public Health on the potential impacts of the transaction on a wide variety of measures and provides a recommendation as to whether the transaction should proceed unimpeded (34). Recent legislation in California has bestowed similar authority and responsibilities to the California Office of Health Care Affordability (OHCA), requiring the agency to conduct intensive reviews of certain transactions and refer concerning transactions to the California attorney general for enforcement (35). Oregon has gone a step further in granting the Oregon Health Authority (OHA) the ability to not only review transactions but also to approve, condition, or deny healthcare transactions after the two-stage review examining a transaction’s potential impact on cost, access, equity, and quality (36).

The last mechanism some states have relied upon to oversee hospital and health system transactions is a more transaction-specific legal mechanism known as a Certificate of Public Advantage (COPA). When a state grants a hospital or health system a COPA it shields the transaction from federal antitrust enforcement under the state action doctrine, which provides immunity to the merging healthcare providers as long as the state clearly articulates its intent to displace competition in favor of regulation and provides active oversight through the attorney general or other state health agency (37). Conditions imposed through a COPA differ from conditions applied by the other authorities discussed above because they require state legislation to authorize, attempt to replace competition with regulation, and mandate intensive oversight by the state throughout the healthcare entity’s existence. While this review did not focus on conditions imposed through COPAs, the evidence of the consequences when COPAs are repealed is useful in illustrating the issue with time-limited conditions when coupled with a lack of broader policies to manage the failures of competition. There has also been concern about the potential for regulatory capture, in which regulators are influenced by the entities they regulate, compromising their impartiality and effectiveness in protecting the public interest (38). Because of these parallels, COPAs can provide insights into the potential shortfalls of conditions imposed by state authorities.

States across the country have used these various legal avenues to impose conditions on healthcare provider transactions. The comprehensive administrative processes laid out in some states, like California and Oregon, can provide for a more consistent and transparent approach to reviewing transactions. This oversight is important not only for state officials to gather information and data on the changes to the state’s healthcare markets that can help inform decisions on future transactions, but also to help ensure that conditions are thoughtfully tailored to substantive concerns and goals and that oversight of those conditions does not waiver with changes in administrations or priorities. Further, assigning responsibility and providing enough resources to a state entity for thorough oversight over imposed conditions is crucial not only to ensure compliance, but also to monitor whether they are achieving their intended effects. The next section will discuss the major categories of conditions and how they have been used to address concerns arising from transactions related to competition, access, quality, and other issues plaguing our healthcare markets.

## 3. Taxonomy of state-imposed conditions

In recent years, states have relied on the legal authorities discussed above to varying degrees to review and subsequently impose conditions on healthcare provider transactions. To identify and examine these conditions, we conducted searches for all 50 states based on the type of legal authorities available in each state and collected publicly available decisions imposing conditions on transactions that were subsequently consummated. To collect conditional approvals from administrative processes, we searched the websites of state attorneys general, CON programs, departments of health, and other healthcare market oversight entities for public postings or other notifications issued between 2012 and 2022 regarding the review and approval of healthcare provider transactions in all 50 states. As we only found nine states with strong public information practices for publishing pre-transactions reviews and decisions, we also conducted online searches for news articles and scholarly works examining healthcare provider transactions to look for any discussion and documentation of conditionally approved transactions. For consent decrees, we also searched the websites of state attorneys general, news sources, and legal databases in all 50 states for consent decrees issued between 2012 and 2022. Our search for consent decrees was limited in many states due to restricted access to state court documents. Because we limited our search for conditional approvals and consent decrees to publicly available information, our data is biased toward states with a commitment to transparency and transactions that garnered media coverage. The lag time between decisions and public availability of the decision may also be a limiting factor, as there may have been conditional approvals that had not yet been published. Nonetheless, we found over 80 decisions imposing conditions from 12 states that formed the basis for our review. As we do not explicitly discuss all the decisions and specific conditions we reviewed, a complete list of the decisions is available as Supplementary material.

After analyzing all available decisions from state attorneys general and state agencies imposing conditions on impending mergers, we found the conditions imposed by state officials generally fall into several major categories, including (1) conditions aimed at managing competition and price, (2) conditions to protect the workforce of hospitals, (3) conditions safeguarding continued and equitable access to services, (4) conditions on financial commitments and investments, (5) conditions to ensure high-quality care, (6) conditions regarding charity care and community benefit requirements, (7) conditions to manage oversight and reporting for compliance, and (8) provisions governing enforcement of the conditions. Table 1 illustrates the frequency in each state of the different conditions and Table 2 provides examples of the types of conditions commonly seen in the different categories across states. Conditions in all categories either require or prohibit certain conduct and are generally in place anywhere from 2 to 15 years.

**Table 1:** Frequency of condition type by state.

State	# of decisions	Competition and price	Workforce	Access and equity	Financial	Quality	Charity care and community benefits	Notice of future changes	Compliance reporting	Independent monitor
CA	14	25%	71%	100%	43%	14%	100%	100%	100%	36%
CT	15	33%	33%	80%	–	27%	67%	60%	93%	53%
ME	6	17%	–	–	–	67%	–	33%	100%	–
MA	5	20%	–	100%	20%	20%	–	40%	100%	20%
MI	4	–	100%	100%	100%	–	100%	100%	100%	100%
NH	8	13%	13%	75%	–	13%	75%	50%	63%	–
NJ	7	–	86%	100%	–	43%	100%	71%	100%	–
NY	1	100%	–	100%	–	–	–	–	100%	100%
NV	1	100%	–	–	–	–	–	100%	–	100%
PA	2	100%	100%	100%	–	–	–	–	–	–
RI	12	–	50%	58%	1%	58%	42%	50%	92%	1%
WA	6	–	–	83%	–	–	83%	17%	100%	–
Total	81	20%	42%	78%	15%	27%	63%	59%	90%	26%

**Table 2:** Examples of conditions imposed.

Competition and Price	Workforce	Access and equity	Financial commitments and investments	Quality	Charity care and community benefits	Notice of future transactions or other changes	Compliance reporting/independent monitor
Prohibit price increases exceeding certain benchmarks Prohibit certain anticompetitive contracting practices, such as tying, all-or-nothing contracting, anti-tiering/steering clauses, most-favored-nation clauses, and exclusive contracting Payers may request separate negotiations or firewalls Prohibit the imposition of system-wide rates unless payer proposes it	Require hospital to retain all or most employees post-transaction Provide notice to state officials of any planned reductions in workforce Prohibit noncompete clauses Prohibit changes to employee benefits Require initiatives for physician employment Maintain privileges for medical staff in good standing	Require that hospital remain open and maintain current licensure Hospital must maintain all or specific set of services at current level Hospital must maintain reproductive care services Require participation in state Medicaid program and/or initiatives to increase number of Medicaid patients May not discriminate based on patient’s ability to pay or payment source Prohibits any form of discrimination Provide culturally and linguistically appropriate services	Require putting aside a certain amount of money to cover operating costs Require a certain amount in capital improvement expenditures at acquired hospital Reinforce existing capital improvement agreements	Must submit annual reports on quality and outcome measures Must implement certain quality of care initiatives Must appoint evaluation team to monitor quality of care Participate in health information exchanges Notify state officials of changes to quality programs Submit a plan detailing how reported savings will be used to improve quality and access Report on improvement in quality outcomes attributable to transaction	Require a minimum spending amount for charity care and/or community benefits Maintain current charity care policy Maintain current community benefit programs Inform patients of charity care and/or financial assistance policies Provide written notice to state officials of changes to charity care policy Provide free or discounted care to low-income patients (e.g., 175% of federal poverty guidelines)	Notify state officials of future transactions Notify state officials of any reductions in services Annual reporting of changes in payer mix and whether it has had an impact on access	Annual reporting on compliance with conditions Appoint independent monitor to monitor compliance Report on implementation of proposed efficiencies from transaction Report on access, quality, and cost of health services Provide annual audited financial statements Report on implementation of certain required initiatives Report on capital investments

### 3.1. Competition

In transactions that have raised competitive concerns, state attorneys general and state agencies have imposed various conditions that either directly or indirectly seek to stem potential anticompetitive conduct enabled by any accumulated market power from the transaction. In response to concerns about post-transaction price increases that ultimately affect patients’ access to affordable care, some states have imposed conditions that directly target prices through provisions such as price or rate restraints, while others address behavior that can indirectly lead to price increases, such as conditions that help maintain fair provider-payer negotiations or prohibit certain contracting practices with payers. This section will review the various types of conditions intended to prevent this behavior and the potential impacts of these requirements.

#### 3.1.1. Price and rate restraints

In response to extensive research that has predominantly found that prices often increase post-transaction, at least four states have attempted to prevent price increases following a transaction by imposing various types of limits on price increases (1, 39). While these price restraints may be effective in preventing the immediate price increases seen post-transaction if accompanied by thorough oversight and accurate data, effectively regulating prices has proven challenging, and experiences from price regulation in COPAs illustrate that using conditions should not be the preferred, long-term solution to managing the price increases frequently seen in consolidating markets (37).

Massachusetts passed legislation in 2012 that established a Cost Growth Benchmark (CGB), a cost containment strategy that sets a limit on the annual increase in the state’s healthcare spending and granted the HPC the authority to monitor and enforce compliance with the benchmark (40, 41). Since then, the state has used the CGB as a ceiling in several instances to limit price increases following healthcare mergers. For example, in 2018, the negotiated consent decree between the Massachusetts attorney general and Beth Israel Lahey Health set an “unprecedented” price cap by prohibiting post-merger price increases from exceeding 0.1% below the state’s CBG for 7 years (42, 43). Massachusetts’ CON program, called the Determination of Need (DoN) program, has also used the state’s CBG as a limit for post-merger price increases, prohibiting the merged entity from increasing prices above the CGB (44). For states that have a CBG, using it as a threshold when instituting price caps can be a relatively straightforward way to set the caps, and having specific mergers conditioned to follow the CBG can add an extra means of enforcement for entities that exceed the benchmark. Furthermore, once conditions expire, the CGB could provide a backstop for excessive price increases moving forward.

Since California has not yet implemented a CGB, the state has used different measures to control post-merger price increases. The California attorney general’s office has imposed conditions in recent transactions that require providers to maintain their existing payer contracts for 5 years and impose percentage caps for annual price increases for any contract renewals during that time. These percentage caps have ranged from 4.8 to 6% for contract renewals (45, 46), although one of these approvals set the cap at 6% for commercial prices and 2.8% for Medi-Cal prices (47). Another conditional approval involving Kaiser Permanente set absolute price caps for Managed MediCal and Medicare contracts for 10 years, in addition to renewal annual increase percentage caps for commercial contracts (48). In all of these decisions, the attorney general’s office is permitted to extend conditions another 3 years and in making that decision shall consider whether the providers have materially violated any of the competition-related conditions. Although these caps protect against price hikes for their lifespan, without broader price regulation policies in place, very little prevents potential increases once the conditions expire.

So far, compliance reporting with these conditional approvals has shown that the restraints have been followed, and little to no empirical evidence exists as to the impact on price after the expiration of these price restraints. There have been multiple investigations, however, into the impacts on price after states repeal COPAs. While COPA price restraints apply to large transactions that significantly impact competition, evidence on the impact of repealing COPA regulations may shed light on the implications of time-limited conditions in other settings. In a policy paper summarizing the results of their five-year COPA Assessment Project, the FTC pointed to several COPA case studies that found that when states repeal COPAs, significant price increases follow (37). For example, one recent study found that for all three COPAs that were repealed prior to 2015, the prices of the previously regulated hospitals substantially increased when compared to controls (49). In light of this research and the frequency with which COPAs are repealed, the FTC has advised states against using COPAs, concluding that hospital mergers with COPA protections often result in unconstrained market power leading to higher prices and lower quality of care in some circumstances (37).

The experiences with COPAs can potentially serve as a cautionary tale about the time-limited nature of most price restraints imposed through conditions. While in place, the state authority responsible for oversight will need recent, accurate pricing data, access to payer contracts, and the expertise to decipher the materials provided to effectively enforce these types of conditions. Ideally, state officials should also be able to modify conditions to respond to changes in healthcare market dynamics, address the effectiveness of the conditions, or extend conditions if necessary. In situations where a time-limited price restraint is imposed, that duration should reflect the time it may take to institute other means of price regulation or pass price regulation legislation (6).

#### 3.1.2. Conditions on healthcare provider-payer negotiations

States have also approached managing potential price increases by imposing conditions on the negotiations between healthcare providers and payers. Recognizing that some transactions lead to an accumulation of market power that may unfairly provide hospitals and health systems with additional leverage, a few states have imposed conditions that dictate the process of these negotiations. For example, the five-year consent decree between Concord Hospital, Inc., LRGHealthcare, and the New Hampshire Consumer Protection and Antitrust Bureau, created a binding arbitration process that is available to payers in the event the hospitals demand unfair rates creating an impasse in negotiations (50). In a different approach, the agreement between the New York attorney general, Faxton-St. Luke’s Healthcare, and St. Elizabeth Medical Center requires the hospitals to negotiate in good faith with payers. If payers believe that the hospitals are acting unfairly, the agreement grants them the right to continue their currently-existing contracts with the hospital for 5 years at current prices (51). Lastly, in two recent decisions from the California attorney general’s office, the conditions state that in the event that the hospitals violate the price restraints or other competition-related conditions, the payer may request separate negotiating teams and firewalls between the transacting hospitals as a remedy (45, 46). Like the price restraints discussed above, provisions such as these can be useful to manage potential price increases for a few years post-transaction, but in the conditions we reviewed, they do not provide avenues for fair negotiations in perpetuity.

#### 3.1.3. Prohibitions on anticompetitive contracting practices

In addition to conditions that can help manage the negotiation process, states have also imposed conditions prohibiting specific anticompetitive contracting terms and practices. While prohibitions on anticompetitive contracting terms and practices have been utilized in consent decrees under antitrust law (52), California’s attorney general office has been at the forefront of prohibiting these anticompetitive contracting practices using their administrative nonprofit review authority. Specifically, decisions have included conditions that prohibit practices that resemble anticompetitive tying, including bans on the use of all-or-nothing clauses, which require a health plan that wants to contract with at least one provider in a health system to contract with all providers in that system—effectively tying all the providers or facilities in the system together at a supracompetitive price (45–48). These conditions also prohibit less extreme forms of tying, such as conditioning the participation of one facility in a payer’s network on the inclusion of one or more less desirable facilities. Additionally, when approving a merger between Methodist Hospital of Southern California and USC Health System, the attorney general imposed conditions that prohibit a practice known as “*de facto* all-or-nothing contracting,” in which a health system sets significantly higher prices or out-of-network fees for its hospitals if the payer chooses not to contract with all of the system’s hospitals (45). Lastly, the attorney general prohibited the inclusion of anti-tiering/anti-steering provisions, which require insurers to place all physicians, hospitals, and other facilities associated with a hospital system in the most favorable tier of providers or at the lowest cost-sharing rate to avoid steering patients away from that network (48). All the decisions prohibiting these practices put these restrictions in place for 10 years, with the option for the attorney general’s office to extend them for another 3 years. These conditions, in theory, have the potential to prevent newly merged entities from overtly leveraging their power to reduce competition and can provide a means of deterrent beyond the threat of an antitrust suit for anticompetitive behavior. That being said, overseeing these types of conditions could be challenging if the state entity charged with oversight does not have access to the contracts or if these terms are not explicitly in the contracts, but are instead negotiated in closed-door negotiations, and the enforcers must rely on payers or other parties to report noncompliance.

When competition-related conditions are time-limited, they may prevent anticompetitive behavior if overseen and enforced effectively but ultimately do not provide a long-term check on any future anticompetitive behavior. When conditions expire, states have the option to threaten or bring an antitrust suit, but that endeavor is lengthy and the outcome uncertain (53). Alternatively, during the time conditions are in place, state legislatures could move to pass legislation that would indefinitely and uniformly prohibit anticompetitive contracting practices or install other price-regulating policies. When a transaction raises competitive concerns, state officials and antitrust enforcers should consider blocking such transactions outright unless there are extremely compelling reasons not to do so.

### 3.2. Workforce

Interest has been growing in the impact of hospital and health system transactions on local labor markets, both in terms of competition as well as ensuring there are enough providers and staff to preserve access to services (54). Hospitals are often the largest employer in a region, and there have been concerns that diminished competition from the acquisition of hospitals and physicians can lead to limited physician employment opportunities as well as significant layoffs of staff post-transaction (55, 56).

When health systems acquire physician groups and then limit the physicians’ ability to work with other hospitals through noncompete clauses or exclusivity agreements, the impact is two-fold. First, physicians are unable to work with other hospitals or health systems or leave that health system unless they are willing to move to another area or even another state (57). Second, competing hospitals that may have previously relied on those physicians to provide services no longer have access to them, which can impact their ability to provide care (58). At least four states have imposed conditions prohibiting non-compete agreements, exclusivity arrangements, and other restrictions on physician employment (17, 18, 50, 59, 60). For example, in 2012, the Maine attorney general entered into a consent decree with MaineHealth after it sought to acquire the only two large cardiology groups in the Portland, Maine area. Conditions included that the cardiologists could not be hindered in their ability to compete or participate in physician networks (59). Around the same time, Renown Health in Nevada sought to acquire two medical practices that provided essentially all the cardiology services in the area. The Nevada attorney general along with the FTC, reached a consent decree with Renown Health to temporarily free the acquired cardiologists from their non-compete agreements to permit them to leave the system without legal repercussions to restore competition for cardiology services (60). In addition to prohibiting non-compete agreements, in the consent decree with LRGHealthcare, the New Hampshire Consumer Protection and Antitrust Bureau also prohibited the use of exclusivity clauses that prevent providers from contracting with other hospitals or health systems (50). The use of noncompete clauses in healthcare in particular has long been an area of contention, with physicians arguing that it hampers career growth and hospitals claiming that noncompete clauses help protect their investment in training physicians and keep services appropriately staffed (61). Although the FTC is currently considering a rule banning noncompete clauses, which would extend to physicians employed by health systems and hospitals (62), restricting these practices through conditions in states that do not already ban them and in situations where they will actively harm competition in a specific market can be a useful tool for states in the meantime.

At least two other states have also imposed conditions to try to monitor and protect the workforce at hospitals post-transaction, specifically against immediate layoffs that impact services and the economic health of an area. In the recent acquisition of two community hospitals in Rhode Island, both the Rhode Island Department of Health and the Rhode Island attorney general imposed conditions to protect the employees of both hospitals. The Department of Health prohibited any layoffs for a year post-transaction (63), whereas the attorney general prohibited any changes to employee benefits for 6 months post-transaction and required that the attorney general’s office must be notified 10 days before there are any reductions to the workforce (64). New Jersey’s CON program also considers the impact of transactions on hospital employees and many of its conditional approvals have reinforced the healthcare entities’ commitment to retaining nearly all employees after the transaction and have required explanations for any workforce reductions (65). Especially in transactions involving financially struggling hospitals and hospitals in rural areas, layoffs may be a strategic step in cutting costs, but state officials should have the ability to closely monitor changes in the workforce to determine whether the layoffs go beyond achieving efficiencies and impact access to quality care for patients.

### 3.3. Access and equity

While consolidation can have troubling consequences for healthcare prices as well as labor markets that indirectly impact access to care, healthcare provider consolidation can also lead to a direct loss of access to services. Conditions geared toward preserving access to care try to address the fact that while mergers and other transactions may provide valuable and necessary resources to hospitals, they can also lead to the termination of less profitable services or even the eventual closure of acquired hospitals (66, 67). Across the states we reviewed, most decisions had provisions intended to protect or promote access. Examples of these provisions include requiring the transacting hospitals to maintain all current services or maintain specific critical services, such as 24/7 emergency departments, maternal care services, or mental and behavioral health services. Additionally, conditions have been utilized to enforce notice requirements for reductions or terminations of services, with some conditional approvals requiring hospitals to provide advance notice to or obtain prior approval from state officials before making any changes to their services (68–70). Identifying essential services and the services most vulnerable to elimination as well as finding opportunities to improve equitable access to care should be a crucial part of any pre-transaction review and approval process. Below, we discuss how states have sought to address specific concerns related to reproductive services, rural health services, Medicaid and Medicare services, access for underserved communities, and cultural and language barriers to access.

Several states have sought to address the loss of access to reproductive health care resulting from transactions involving Catholic hospitals, which must adhere to the Ethical and Religious Directives for Catholic Health Care Services (ERDs) (71). The ERDs establish the ethical and moral boundaries within which care providers must operate, specifically forbidding providers in their health systems from providing various reproductive health services, including abortion, contraception, and tubal ligation. In 2019, as a condition for approving the vast merger between Dignity Health and Catholic Health Initiatives creating CommonSpirit, the California attorney general required that the hospitals in California continue reproductive health services for 5 years, and for an additional 5 years, notify the attorney general prior to eliminating those services (70). The New York attorney general has also included provisions protecting access to reproductive care services in the negotiated consent decree governing the affiliation between Faxton-St. Luke’s Healthcare System and St. Elizabeth Medical Center (51). The growing footprint of Catholic hospitals across the country has raised further access concerns regarding gender-affirming and end-of-life care services in the secular hospitals and health systems they acquire (72, 73). As access to services that contravene the Catholic ERDs becomes more constrained across the country, state enforcers and regulators can protect these services by imposing access-oriented conditions more frequently and for longer periods of time.

Hospital transactions in underserved and rural areas, in particular, often disproportionately impact access to care (66). Rural hospitals face a double-edged sword when it comes to consolidation: while transactions like mergers and acquisitions may bring in an influx of capital and improved infrastructure, often the acquirer opts to eliminate services as a cost-savings strategy, frequently targeting those that are duplicated, less profitable, or have lower reimbursement rates (66). For those in rural areas with an already limited pool of healthcare options, the elimination of these services can delay access to emergency treatment, increase treatment costs, and cause patients to forego care altogether (74). The discontinuation of services, such as emergency departments and labor and delivery units, also contributes to worse health outcomes, more expensive care, and exacerbation of already problematic inequities in care (75, 76). Utilizing conditions like those mentioned above is crucial to preserving access to services until another long-term solution can be found to protect access in these areas.

In addition to requiring the continuation of services, some states have also taken steps to encourage more equitable access to services. For example, requiring hospitals to continue or increase their participation in Medicaid and Medicare can be particularly impactful in rural and underserved areas, where access to care is an issue both in the number of available providers and the distance patients must travel to seek care (77). Both the Massachusetts attorney general and the DoN program have imposed conditions requiring provider participation in MassHealth (Massachusetts’s Medicaid program) and the State Children’s Health Insurance Program on the merger creating Beth Israel Lahey Health after the HPC found that the inpatient Medicaid mix would be among the lowest of the major health systems in eastern Massachusetts (43, 78, 79). Specifically, the consent decree negotiated by the attorney general guarantees that there will be no caps on MassHealth patients and requires the health system to create a new program to increase the number of MassHealth patients (43). Examining the patient mix at transacting hospitals and the demographics of the communities they serve can be an important step in identifying opportunities to improve access for these populations.

State officials have also identified opportunities to ensure healthcare providers meet the needs of the various communities they serve. At least two states, California and Massachusetts, have required the transacting systems to make financial commitments to bolster access and support community entities providing services for low-income and underserved communities (43, 70, 80). For example, in the agreement with Beth Israel Lahey Health, the Massachusetts attorney general received a $71.6 million commitment from the health system that provides financial support for community health centers, safety net hospitals, and behavioral health access (43). State officials have also included provisions that aim to make sure hospitals and health systems hear the voices of community members moving forward. For example, California has required transacting hospitals to maintain a community board that must be consulted prior to changes to services or community benefit programs (44, 46, 47), while Connecticut often requires community members to be placed on the hospital boards (81–83). Conditions like these, which are designed to maintain and grow access to healthcare services for underserved communities, can grant states time to develop alternative options for those populations after the conditions expire or after the initial financial commitments have been made.

In addition to the challenges with access to healthcare services, many patients experience significant language and cultural barriers that compromise their care (84). These barriers create extremely challenging circumstances for some people seeking to avail themselves of the healthcare system, making it difficult for patients to communicate their symptoms, ask questions of clinicians, or understand provider instructions, which in turn impedes the abilities of providers to administer appropriate patient care (85). In recognition of how language and cultural barriers impact health equity, California, Connecticut, New Jersey, Rhode Island, and Washington have imposed requirements for hospitals to address access to language services that often reiterate existing laws. While there are laws and regulations at the federal level requiring similar policies (85), these conditions can help reinforce those requirements.

Many of the conditions described in this section respond to concerning trends across the country involving increasingly shrinking access to services. While conditions can serve as a stopgap solution to preserve access, in most cases, they do not offer viable, long-term solutions, especially for hospitals facing increasing financial troubles that would benefit from broader and more permanent policies designed to protect the access to health care communities rely on.

### 3.4. Financial commitments and investments

While conditions that protect and enhance access to services are critical, going a step further and imposing conditions aimed at protecting the continued operation of financially struggling hospitals can be a more challenging task. One way is for conditions to reiterate the transacting parties’ agreement to provide capital commitments post-transaction. For example, the California attorney general’s office has reiterated transacting health systems’ investment commitments going toward improving facilities (86, 87). Including promises set out in the agreement between the parties in the formal conditions may enable the attorney general to enforce provisions in the agreement that may have otherwise been difficult to oversee or enforce.

In our sample, two state attorneys general went a little further and imposed conditions on transacting providers that required either investing or setting aside finances to ensure that the community received the promised benefits of the transaction. In 2015, the California attorney general’s office took a more involved approach in the decision conditionally approving BlueMountain Capital Management’s, a private equity firm, acquisition of Daughters of Charity Health System, saving the failing health system from bankruptcy (88). To ensure that the health system would actually be financially supported moving forward, the California attorney general’s office required that $180 million be invested in capital improvement expenditures at the health systems facilities as part of the conditional approval of the deal (88). More recently, the Rhode Island attorney general took unique steps to safeguard the continued operation of Roger Williams Medical Center and Our Lady of Fatima Hospital, two local safety net hospitals, owned by Los Angeles-based for-profit Prospect Medical Holdings (PMH) (89). In the proposed transaction, a private equity group effectively wanted PMH to buy out their existing ownership stake. The Rhode Island attorney general was concerned that this transaction would contribute to PMH’s increasingly vulnerable financial state and would threaten the viability of the two hospitals that are financially dependent on it (90). To protect the two hospitals from PMH’s troubling business practices, the attorney general required several financial commitments from the transacting entities, including requiring the transacting entities to put $80 million in an escrow account that can be drawn upon to cover the two hospitals’ operating and capital expenses if Prospect fails to fulfill its financial obligations (64). The attorney general also required that any transfers of assets or encumbrances must undergo prior approval by the attorney general for 5 years. Although these types of protections are only in place for a few years, keeping hospitals operational for a while is a better outcome than them closing in that time.

### 3.5. Quality of care

While maintaining access to affordable care is crucial, states have also imposed conditions intended to monitor that hospitals deliver on promises to maintain or improve the quality of that care post-transaction. While hospitals and health systems frequently claim transactions will serve patients’ interests by improving the quality of care, studies have found that the assertion that transactions improve quality is often unsubstantiated (91–93). For instance, Nancy Beaulieu and colleagues at Harvard University analyzed the quality impacts following 246 hospital acquisitions by health systems and found modestly worse patient experiences and no significant changes in readmission or mortality rates (91).

To monitor the quality of care after mergers and to ensure patient care is not compromised in the interest of other priorities, some states have imposed conditions to hold hospitals accountable for post-transaction quality of care. Maine has required annual reports on quality improvements and outcome measures for 3 years in nearly all its CON conditional approvals (94). Rhode Island has addressed post-transaction quality by requiring hospitals to increase patient enrollment in CurrentCare, a platform that provides real-time patient information as well as data on various quality, efficiency, and safety measures, and to implement quality improvement initiatives, including launching programs to prevent unnecessary hospital admission and readmissions and to improve screenings for alcohol abuse (95–97). Monitoring quality over time is critical to understanding how transactions impact patient outcomes and conditions that require transacting hospitals to provide data on patient experience, quality of care, and patient outcomes can be a useful tool to allow that state to identify trends and changes.

### 3.6. Charity care and community benefits

In addition to many of the conditions mentioned above, transactions involving nonprofit hospitals require additional considerations and conditions unique to nonprofits. Unlike their for-profit counterparts, nonprofit hospitals are expected to provide community benefits, including charity care (free or discounted care) in exchange for exemptions from local, state, and federal taxes (98). Recently there has been a growing spotlight on the behavior of nonprofit hospitals and significant questions as to whether they are fulfilling the responsibilities tied to their tax-exempt status (99, 100). In fact, recent research has shown that nonprofit hospitals devote a smaller or similar share of their operating expenses to charity care than for-profit hospitals (101). Factors contributing to this trend likely include the lack of clear requirements and consistent oversight from federal and state officials (102, 103). At the federal level, the IRS does not specify quantitative measures for charity care or community benefits, while state requirements and oversight vary widely across the country (102, 104).

Recognizing these shortcomings and the importance of nonprofits’ obligations to the communities they serve, many of the states we reviewed, including California, Connecticut, Maine, Massachusetts, New Hampshire, New Jersey, Rhode Island, and Washington have used their approval authority to impose conditions intended to protect against any reductions or eliminations in charity care or other community benefits post-transaction. Conditions addressing community benefits generally included conditions such as requiring hospitals to maintain their current charity care policies, notifying the appropriate state officials of any intended changes to the policies, requiring hospitals to notify patients of the availability of financial assistance or post those policies publicly, and requiring hospitals to maintain or implement community benefit programs that address population health and social needs.

Going a step further, the California attorney general’s office through its nonprofit review has set required minimum amounts hospitals must spend on charity care and community benefits for several years post-transaction in virtually all conditional approvals (105). In 2018, multiple hospitals petitioned the California attorney general to significantly reduce these obligations in light of the Affordable Care Act making health insurance more widely available (106). The attorney general denied these requests and mandated that the hospitals immediately pay the outstanding amounts of their obligations under the conditions to tax-exempt entities providing direct medical services to the community in the hospitals’ service areas for failure to meet their charity care requirements in the previous years (106). Conditions that impose minimum charity care or community benefits requirements can set meaningful and enforceable standards for nonprofits to follow and create a means of enforcement in the absence of state or federal law should the hospitals fall short of these obligations to the communities they serve.

### 3.7. Post-transaction oversight

For any of the conditions discussed above to be meaningful, there must continuous and in-depth oversight to hold hospitals and health systems accountable to their obligations. Effective oversight requires two components: (1) hospitals or health systems need to provide recent and accurate data illustrating compliance, and (2) states need the resources and expertise to analyze that information.

First, the state conditional approvals and consent decrees we reviewed impose various reporting requirements to demonstrate compliance for the duration of the conditions. For example, in all conditional approvals in California and Connecticut, hospitals must submit annual reports detailing their compliance with the conditions imposed. Similar requirements have also been set in statutes and regulations, rather than in the conditions themselves, as seen for several states’ CON programs, including Massachusetts (107), Maine (108), and Washington (109). As part of compliance reporting, states have also required healthcare entities to submit information to help the oversight entity monitor other potential changes that arise from the transaction. This information can include detailed financial reports, healthcare quality reports, and reports on the efficiencies achieved as part of the merger. Reporting requirements provide the state with valuable information with which to verify compliance and assess the impacts of the merger. Relying only on self-reported data provided by the monitored healthcare entities, however, leaves open the possibility that only the most favorable data will be disclosed, data may be incomplete, or it may not fully reflect the realities of operations. Therefore, state officials should consider taking steps to confirm the validity and accuracy of the submitted information.

Second, states need to devote significant time, funding, and expertise to effectively analyze and assess compliance. While state agencies and attorney general offices are responsible for this task, some have found ways to minimize that burden. States including California, Connecticut, Massachusetts, and Michigan have utilized third-party independent monitors at the expense of the healthcare entities to continuously assess compliance and outcomes post-merger. The use of an independent monitor may also have the added benefit of minimizing actual or perceived bias in the oversight process, thereby increasing confidence in the findings. The efficacy of an independent monitor greatly depends on who picks the monitor and who the monitor is. Because their role is to assist state officials in overseeing conditioned transactions, the state should select the monitor. Leaving the decision to the transacting entities allows them to pick a monitor who may not be objective or will not provide thorough oversight. Furthermore, because of the specific expertise and experience required to fully understand and evaluate the intricacies of healthcare finances, operations, facilities, investments, and staffing, some of those most qualified to conduct compliance reviews may also have ties to the industry, potentially compromising their objectivity. Therefore, independent monitors less closely tied to the industry, such as academics or retired professionals, may be better suited for this role.

Oversight can serve as not only a check on adherence to conditions but a mechanism for deterrence, discouraging entities from ignoring their obligations. By failing to hold entities accountable to the goals of reducing costs, maintaining competition, and promoting access, equity, and quality, the power of conditions in future approvals becomes significantly weaker. States should only approve transactions with conditions if they are equipped and committed to monitoring and oversight.

### 3.8. Enforcement

Reporting and oversight can only hold parties accountable if there are consequences for noncompliance. Enforcement provisions without appropriate remedies or penalties are neither a deterrent nor a punishment. The remedy chosen should depend upon the entities, the infraction, and the state’s goals. Many state officials first address noncompliance by offering to facilitate compliance or remediate improper conduct, such as requiring entities to submit performance plans, refund excess revenue generated through noncompliance, or require funding for health priorities or community benefit programs. For example, the Pennsylvania attorney general will first provide a reasonable opportunity for the entity to cure its noncompliance with consent decrees within 60 days and may take remedial action thereafter (17, 18). In its consent decree with MaineHealth, the Maine attorney general required that if the entities violate the conditions relating to rate restrictions, they shall refund 110% of excess revenue generated from non-compliance to affected commercial payers (59). Per Massachusetts DoN regulations, the DoN program may require the noncomplying healthcare entity to fund projects which address one or more of the established health priorities for instances of non-compliance (110).

Several states also require the entity to reimburse enforcement actions that seek to hold them accountable. The Michigan attorney general’s office has required providers to allocate a certain amount of money to cover the attorney general’s expenses for non-compliance enforcement actions if the entities do not remedy the violation within a given amount of time (111). By law, the California attorney general is entitled to recover attorney fees and costs incurred in remedying each violation of a condition of approval (112). Holding the transacting providers responsible for the costs of enforcement in light of continued noncompliance, not only eases some of the burden on state resources but also can serve as a deterrent for noncompliance.

Enforcement, however, is not necessarily a straightforward endeavor. In 2019, as part of the conditions granting a CON for the merger of two health systems creating Nuvance Health, the Connecticut Office of Health Strategy (OHS), required the merged entity to annually attest that they have maintained certain services as well as to maintain sufficient staffing for OB/GYN services at Danbury, Norwalk, and Sharon Hospitals (82). Within 2 years, however, OHS received complaints alleging that Sharon Hospital was not in compliance with these conditions, leading the agency to launch an investigation into the alleged noncompliance after receiving conflicting information from the hospital (113). In the midst of the allegations of noncompliance, the provider applied for a subsequent CON to formally allow Sharon Hospital to terminate its labor and delivery services despite the conditions previously imposed, citing declining birthrates and challenges attracting and retaining staff to support maternity services (114–116). In response, there has been significant pushback from the community as well as other state officials against the termination request (116, 117). In particular, the Connecticut attorney general’s comment to OHS regarding the termination request aptly outlines how the closure will impact patients, particularly Medicaid and other low-income patients, which comprised 48% of the hospital’s post-birth discharges in 2021, and how terminating these services will erect barriers to access for those patients (117). OHS’s noncompliance investigation and its consideration of the new request to terminate services both appear to be ongoing, however, they illustrate the difficult position state officials might find themselves in when providers attempt to circumvent conditions that are responsive to community needs, claiming that they threaten financial sustainability. Despite these claims, providers must still be held responsible for noncompliance and should not be able to inappropriately deprive patients of accessible care. Circumstances like the one in Connecticut also shed light on the need for better policies beyond conditions that can help hospitals that are truly struggling without compromising access.

Because effective oversight and enforcement of conditions are complicated, time-consuming, resource-intensive, and sometimes may ultimately prove ineffective, it is important to distinguish between deals that can serve the interests of the community but need oversight, from transactions that pose significant risks such that, even with conditions, monitoring, and enforcement, they may do more harm than good. Making this determination is dependent on a variety of factors, such as size, hospital type, financial status, geography, market landscape, pricing practices, and community needs. For example, a conditional approval of an acquisition that will prevent a small struggling rural hospital from shuttering, can not only help keep the hospital open but can, in theory, also protect against the loss of critical services for the community. In contrast, for a merger that, due to its size or accumulated market power, is likely to further diminish competition and lead to anticompetitive price increases, conditions are hardly going to replace the resulting loss of competitive forces and will just leave the market more consolidated. As the consensus is that it is difficult to “unscramble the egg” after a transaction has occurred (118), even in situations where conditions are likely useful, state officials should consider the potential consequences of a healthcare provider’s failure to adhere to the conditions, what may result after the expiration of the conditions, and how the state can use the time the conditions are in place to prepare other policies or solutions to prevent any negative outcomes in the long-term. The next section delves into the shifting perspectives on the use of conditions, the specific situations when states should consider imposing conditions, and the steps to achieve useful and effective conditions.

## 4. Considerations for states

Although it is relatively common for states to impose conditions on hospital and health system transactions, there is notable skepticism about the use of conditions as a remedy when a transaction raises competitive concerns among federal antitrust enforcers, courts, and scholars. Critiques include evidence from other industries that conditions generally have not achieved their intended purposes, that they “risk excessive government entanglement in the market,” and ultimately still allow markets to consolidate and entities to accumulate market power (119–124). Alongside these concerns about the use of conditions, there has also been a shedding of a long-held presumption that many hospital transactions create sufficient efficiencies to warrant them to proceed unimpeded except in particularly egregious cases of consolidation (125). State officials should consider these shifts as well as the challenges of oversight and enforcement when considering conditionally approving hospital deals.

In light of these changing attitudes and the well-documented detrimental effects of hospital consolidation, state review should carefully consider the long-term consequences of permitting a transaction to proceed with conditions and how necessary it is to permit the transaction to move forward. While there are situations in which conditions are the best outcome, there are many situations in which they are not, especially when the transaction raises competitive red flags. The clearest situation in which imposing conditions can provide a positive outcome is in a transaction that prevents a hospital from closing and preserves the community’s access to care. Rural hospitals are often vulnerable and have been closing at an alarming rate. Since 2010, over 100 rural hospitals have closed and another 89 have significantly reduced services, leaving healthcare deserts across the country (126, 127). In these cases, the repercussions of a hospital closing are considerably worse than imperfect conditions, which can be used to at least keep the hospital operational for a certain period, giving the state time to develop alternative means of accessing care for the impacted population.

Conditions may also be an appropriate route to take for transactions that would have otherwise escaped any sort of scrutiny and would be challenging to block under existing federal or state law, yet raise competition concerns. These transactions include those that do not trigger federal antitrust review, such as smaller transactions, and those that would be difficult for state officials to challenge under state or federal antitrust law, such as those that do not fit neatly into the traditional horizontal merger antitrust analysis (e.g., cross-market or vertical mergers). The relatively high threshold for federal antitrust review creates a chasm through which potentially problematic transactions can fall without state-based review processes to catch them. For instance, research has shown how “stealth consolidation,” or the accumulation of seemingly innocuous individual transactions, can lead to damaging effects on markets (128). These risks are why state pre-transaction review and approval processes are so important and why it is essential that legislatures grant state officials broad authority to review and subsequently block or condition transactions on grounds that impact access to affordable care. When blocking a transaction is not an option, imposing conditions is the next best option as long as there are clear goals as to what the state or the providers should accomplish in the time frame of the conditions. Conditions should aim not only to prevent harm but also to ensure that the proposed benefits are realized as well.

In most other circumstances when the impact on competition is a concern, state officials should err on the side of taking steps to block the transaction. The status quo of permitting most transactions to proceed has left most healthcare markets across the U.S. consolidated, leaving too many without access to quality and affordable health care. Competitive forces are simply insufficient to restrain prices in most markets, so even small transactions that typically go unnoticed by federal antitrust regulators can be harmful. If a potential deal would require multiple conditions to safeguard state priorities, the more effective strategy is likely to block the deal outright, rather than attempt to address the negative effects through conditions that require a heavy commitment of time, resources, and staff and that may ultimately not mitigate the harm caused by the transaction or, at best, provide just a temporary delay. A stronger stance against consolidation is desperately needed to preserve the competitive markets we have left.

### 4.1. Recommendations for imposing conditions

In situations where permitting a transaction to proceed with conditions remains the best option, several broad considerations should be taken into account. First, the conditions should be transaction-specific and thoughtfully tailored to address the precise areas of concern raised during the review process and address likely responses to the conditions by the hospital. For example, if as a condition of approval, the hospital must keep certain services available, a price restraint might also be prudent to prevent any price hikes on those services. This tailored application of conditions requires an in-depth review of the transaction that examines how the transaction will impact prices, access, equity, and quality, and should include gathering and considering input from the community, physicians, nurses, and other hospital staff, through public comments and holding public hearings. This process takes time and resources and states like Massachusetts, California, and Oregon can rely on outside sources to provide in-depth reports and pinpoint areas of concern. In Massachusetts, the HPC conducts Cost and Market Impact Reports (34), whereas the California attorney general has utilized academics or other consultants to produce reports on the potential impacts of proposed transactions (25, 105). These publicly available reports from both states have identified potential negative impacts from transactions that have subsequently informed the conditions imposed. In a different approach, Oregon law permits the OHA to convene a Community Review Board to help review transactions, which must include members from the communities affected by the transaction (36, 129). The Board then provides non-binding recommendations to the OHA as to whether to approve, condition, or block the transaction (36).

Second, if they have discretion, state officials should carefully consider the length of time conditions are imposed, what goals the state can accomplish in that time, and the lasting impacts of imposing conditions for that length of time. States should consider enabling the reviewing entity to modify or lengthen conditions if market conditions have changed or if conditions have not been followed. While conditions imposed through administrative processes are often time-limited by law, conditions in consent decrees can theoretically last in perpetuity, but practically that may be challenging to achieve and oversee. Although longer-term conditions can be useful to achieve and sustain state priorities, years of monitoring can be burdensome, requiring a significant expenditure of time, resources, and personnel. When possible, targeting longer-term conditions on the areas with the most significant risk may prove to be more efficient and effective, directing resources where they can have the most impact. In other cases, states should consider whether imposing conditions for a limited period would enable the state to pass legislation, attract another entity to provide services in the area, or otherwise promote competition in the area. That being said, if it seems likely that the imposition of conditions would only serve to delay the adverse outcomes, not prevent them by providing time and resources for more permanent changes, states should reconsider approval or face the reality of overseeing long-term conditions.

Third, while certain conditions can temporarily fill existing healthcare policy gaps, they should not be relied upon in lieu of broader policies and passing comprehensive healthcare legislation. But as these policies make their way through the legislative process, conditions may be an acceptable way to impose those policies on particular entities, before they are enacted state-wide, and use the experience of overseeing and enforcing those conditions to inform future legislation. There may also be less political opposition to imposing conditions compared to passing certain kinds of legislation, such as strong policies for price regulation. Using conditions in this way can also raise public awareness of the need for legislation, potentially provide proof of efficacy, or provide a means of enforcement before a law is passed. For example, if a state is considering passing legislation banning anticompetitive contracting practices like all-or-nothing or tying, punishing a health system for noncompliance with conditions prohibiting those practices may be more efficient and effective than the attorney general having to pursue an antitrust suit, such as the long and resource-intensive state case against Sutter Health (130).

Fourth, state officials need a deep understanding of both the state and local healthcare markets as well as the likely impacts of the transaction to target conditions to the current market conditions. Information such as how consolidated a market is already, current market dynamics, preexisting quality concerns, the patient mix at neighboring hospitals, and recent changes to available services should inform the conditions imposed. Having a state agency, like Massachusetts’s HPC, that tracks and can provide credible data analysis on healthcare cost and quality measurements can immensely help with this endeavor. However, access to robust data is still something states are grappling with, even those with an all-payer claims databases (131). Although not comprehensive, conditions that require extensive reporting could begin to supply information that could be used to inform future conditions or larger policy initiatives.

Fifth, compliance requirements should mandate that the transacting hospitals demonstrate not only that they are complying with any conduct restrictions, but also provide substantial and explicit evidence and data that they are achieving any purported efficiencies, cost-savings, or other alleged benefits and how those benefits are improving access and quality of care. Further, any instances of noncompliance with any of the requirements should be met with sufficient penalties to serve as effective deterrents. Beyond hefty fines, provisions requiring other remedies could also be included within the approval decision or mandated in statutes or regulations. Examples include the California attorney general specifically requiring hospitals that do not comply with charity care requirements to pay those deficiencies directly to local nonprofits providing direct medical services and the Massachusetts DoN program regulations requiring noncomplying entities to spend an amount equal to a certain percentage of the transaction cost on DoN program-approved projects (106, 110). Conditions are relatively meaningless if they are not enforced and do not ensure that the approval benefits the community.

To achieve all these recommendations, state officials must have sufficient oversight authority through the attorney general’s office, CON program, or another state agency to conduct in-depth reviews of a wide range of transactions and the flexibility to approve, reject, or condition the transaction to address concerns on various fronts. State legislatures need to make sure that new laws granting authority or amendments to existing authority allow state officials to impose meaningful conditions without being accused of overstepping their authority. State officials should be given broad enough authority to adapt to changing trends in how healthcare deals are structured and address concerns unique to certain transactions, such as the involvement of private equity in the healthcare space (132).

### 4.2. Potential provider responses to conditions

In cases where strong conditions are the best path forward, it is also important to recognize that state officials may face significant resistance from health systems and hospitals. The imposition of strict conditions has led to entities abandoning transactions (133), and while this can be a positive outcome if the transaction would have led to more consolidation without many distinct benefits, state officials should be aware that some entities may be prepared to let the deal fall through to avoid conditions, even if that means letting a hospital close. For example, the California attorney general imposed relatively modest conditions intended to protect basic services for the largely uninsured and Latino-majority community on the acquisition of Madera Community Hospital. In response, the acquirer Trinity Health, a nonprofit Catholic health system, pulled out of the deal, ultimately leaving Madera without much-needed financial help and forcing it to close (134, 135). The closure has meant the loss of the only facility providing adult emergency services in the county (135). Situations like this one illustrate the need for states to have more policies at their disposal to prevent the closures of hospitals providing much-needed services, such as global budgets and other policy options geared at helping struggling hospitals (136, 137). While an acquisition may be the most appropriate solution in some cases, that should not dissuade state officials from imposing strong conditions on already powerful health systems.

Transacting providers may also challenge the conditions imposed through administrative approval processes. In 2020, in the first transaction the California attorney general utilized conditions to address competitive issues, the hospitals sued the attorney general claiming that imposing those types of conditions was outside the attorney general’s nonprofit hospital approval statutory authority (138). The case was ultimately settled with slight changes to the competitive impact conditions, but the challenge illustrates a potential hurdle facing state officials wanting to impose types of conditions that they have not previously used (46). While agency decisions reached through administrative processes are generally given deference in court, having to defend those decisions in court takes up valuable time and resources (11). The California attorney general has since imposed similar conditions on other transactions without issue, illustrating that while there might be initial pushback, setting that precedent for using transaction oversight authority to its fullest extent is a worthwhile endeavor (105). Despite being limited to transactions involving nonprofits, California’s attorney general has a relatively broad authority to review transactions for a variety of factors, including competition, access, quality, as well as factors specific to nonprofits, and impose conditions relating to each of those categories, but not all state officials have been granted that same breadth of review (13). For conditions to target all potential impacts on the market, state officials need broad statutory authority to address a wide range of concerns and the means to track and enforce conditions throughout their existence.

## 5. Conclusion

Being able to utilize conditions is an important tool for many states to ensure hospitals and health systems deliver on the promises of the benefits of transactions and that they continue to serve their communities. The use of conditions, however, should be carefully considered when a deal raises competitive concerns and threatens the last vestiges of competitive forces we have left. Any time state officials impose conditions, they should do so with an eye toward future policies and with the intention of fulfilling concrete goals that will defend or improve access to affordable care for years to come.

## Author contributions

AM, KG, and JK contributed to the conception of the work. AM and RD collected research and drafted the manuscript. All authors participated in substantially editing and revising the manuscript critically for important intellectual content and have read and approved the submitted version.

## Funding

This work was supported by Arnold Ventures.

## Conflict of interest

The authors declare that the research was conducted in the absence of any commercial or financial relationships that could be construed as a potential conflict of interest.

## Publisher’s note

All claims expressed in this article are solely those of the authors and do not necessarily represent those of their affiliated organizations, or those of the publisher, the editors and the reviewers. Any product that may be evaluated in this article, or claim that may be made by its manufacturer, is not guaranteed or endorsed by the publisher.
